# Understanding of Information about Medicines Use among Parents of Pre-School Children in Serbia: Parental Pharmacotherapy Literacy Questionnaire (PTHL-SR)

**DOI:** 10.3390/ijerph15050977

**Published:** 2018-05-03

**Authors:** Stana Ubavić, Nataša Bogavac-Stanojević, Aleksandra Jović-Vraneš, Dušanka Krajnović

**Affiliations:** 1 Medicines and Medical Devices Agency of Serbia (ALIMS), 11221 Belgrade, Serbia; 2 Department of Medical Biochemistry, Faculty of Pharmacy, University of Belgrade, 11221 Belgrade, Serbia; 3 Institute of Social Medicine, Faculty of Medicine, University of Belgrade, 11000 Belgrade, Serbia; 4 Department of Social Pharmacy and Pharmaceutical Legislation, Faculty of Pharmacy, University of Belgrade, 11221 Belgrade, Serbia

**Keywords:** parents, pharmacotherapy, health literacy, health education, pre-school children

## Abstract

Parental health literacy plays an important role in children’s health. Experiences from pharmacy practice show that is necessary to check if parents understand instructions about use of medicines for children. This study aimed to assess pharmacotherapy literacy of parents of pre-school children and to examine association of parental pharmacotherapy literacy level with parent’s socio-demographic characteristics. The study was cross-sectional, conducted among parents of pre-school children (1–7 years of age), in kindergartens in several municipalities of Belgrade, Serbia, during regular parents meetings, from May to October 2016. Functional health literacy was measured by the Serbian version of the Short Test of Functional Health Literacy in Adults (S-TOFHLA). Parental pharmacotherapy literacy was assessed with newly constructed PTHL-SR questionnaire with good psychometric characteristics (Parental pharmacotherapy literacy questionnaire—Serbian). Overall, 813 parents participated in the study, mostly females (81.30%), between 30 to 40 years of age (70.85%) with two children (56.70%). Almost all of our study participants (99%) had adequate health literacy as assessed by S-TOFHLA. Mean score on PTHL-SR was 72.83% (standard deviation was 13.37), with better results among females than males (72% of women were in the group of highest PTHL-SR results). Our study showed that many parents (76.5%) knew the appropriate usage of non-prescription medicine for children, 57.2% parents were able to correctly calculate the dose of oral syrup for a child, and only 43.3% were able to interpret non-prescription dosage information written on the package. The majority of parents (61.3%) would make a dosage to child based on age and not on their weight. Every fifth parent with adequate functional health literacy measured by S-TOFHLA test, achieved the lowest results measured by PTHL-SR. Higher performance of the PTHL-SR was significantly correlated with education (*p* < 0.001), female sex (*p* < 0.001), married parents and those living in common-law (*p* < 0.001), older parents (*p* < 0.05) and parents who have more children (*p* < 0.05), and are non-smokers (*p* < 0.05). These results provide evidence that limitations in understanding common information about use of medicines are widespread among parents of pre-school children and encourage efforts for further investigation. PTHL-SR questionnaire may be a useful tool for identification of parents who need more instructions and assistance from healthcare providers, above all in providing better communication, written or spoken at community pharmacy settings.

## 1. Introduction

Health literacy is the term which has been used for more than 40 years in the scientific literature to describe the relationship between patient literacy levels and their ability to comply with prescribed therapeutic regimens [1]. It means applying literacy and numerical skills to health related materials such as prescriptions, appointment cards, medicine labels, and directions for home health care [2]. Health literacy is related to context [3,4], and in the pharmacy setting, can greatly impact the safe and effective use of medicines [5]. Poor health literacy may include misunderstanding of written medicine instructions, inadequate adherence to prescribed regimens, and inability to follow advice from health professionals regarding side effects and possible contraindications [5,6]. Individuals with limited health literacy experience difficulties in understanding drug labels [6]. Even educated patients face problems interpreting labels and patient information leaflets, as these tasks require understanding and application of information [5], thus emphasizing that higher education is not a requisite for understanding medical information [7]. These facts highlight the need for defining a pharmacotherapy literacy as “an individual’s capacity to obtain, evaluate, calculate, and comprehend basic information about pharmacotherapy and pharmacy related services necessary to make appropriate medication-related decisions, regardless of the mode of content delivery (e.g., written, oral, visual images and symbols)” [8].

In the pediatric population, poor pharmacotherapy literacy of parents can cause serious problems in treatment. A study conducted in France [9] showed that parent’s knowledge and practices about use of medicines for treatment of fever differ from recommendations in patient leaflets and without consulting a pediatric or pharmacist. This emphasizes the fact that parents are frequently the ones deciding on the type of medicine administered to a child, especially in emergency situations [9].

Moreover, oral antibiotic medicines were incorrectly prepared and reconstituted by parents in about 50% of cases, resulting in a risk of overdose or underdose [10]. According to a recent study in Australia [11], almost half of parents could not accurately determine weight-based doses. Errors in medicine dose or frequency of dose to children occur very often in cases where adults are preparing the medicines [12].

It was found that the format and content of labels could be improved in order to help parents understand the information about medicines [13]. These studies implicate parental pharmacotherapy literacy as one of the key factors influencing incorrect medicine administration, wrong dosage delivery, and miss-timed medicine delivery [14]. 

Researchers in parental and caregiver health literacy have mostly focused on asthma, nutrition, diabetes, and children with special care needs [14,15,16,17]. 

In the study of Kumar et al. [18], parental health literacy and numeracy skills needed for understanding instructions related to nutrition, injury and preventive care of young children were assessed using the Parental Health Literacy Activities Test (PHLAT). 

Studies that assessed health literacy among parents and caregivers of children were not specific for assessing their pharmacotherapy literacy related to use of medicines for pediatric population [19]. 

Stilley et al. developed and examined psychometric properties of a Medication Health literacy measure tool, similar to Newest Vital Sign, in order to assess health literacy related to understanding and using information on prescription medication labels [20].

Furthermore, there were no studies that examined pharmacotherapy literacy of parents of pre-school children in Serbia, or understanding of information about use of medicines in the pediatric population. The previous study in Serbia by Jovic-Vranes and Bjegovic-Mikanovic evaluated health literacy among primary care patients using S-TOFHLA and TOFHLA as instruments for assessment of health literacy [21,22]. S-TOFHLA showed a “ceiling effect” [23] however was not sufficient to characterize pharmacotherapy literacy. 

Our aim was to investigate knowledge and understanding of information together with numerical skills about medicines among parents of pre-school children with a newly constructed and validated Parental pharmacotherapy literacy questionnaire written in the Serbian language (PTHL-SR) [24].

## 2. Materials and Methods

### 2.1. Study Population

This cross-sectional study was conducted among parents and caregivers of pre-school children (from 1 to 7 years old) in a few municipalities of the city of Belgrade, Serbia. The study populations were both male and female, at least 18 years old, living as a parent, caregiver or a legal representative, or in a close relation to a child, and speaking the Serbian language. Selection of kindergartens was done on a random basis, taking into account kindergartens from municipalities with highest and lowest average monthly salaries. The number of respondents for the sample was calculated based on number of children enrolled in kindergartens in the city of Belgrade. We calculated sample size for a simple random sampling plan without replacement [25]. To determine the sample size we used a 3% margin of error and a 95% confidence level. The proportion of a sample that will choose a given answer to a question was estimated at 72.5% according to the results from Jovic-Vranes and Bjegovic-Mikanovic study which examined health literacy in the Serbian adult populations [21,22]. The Finite Population Correction factor was used for the final sample size calculation. The population size of pre-school children in year 2013 in Belgrade was 98,207 [26]. Calculated sample size in this study was 844 but after inclusion of response rate of 70% the total final number of examined parents were 1205 with the expectation of receiving 844 surveys back from this sample.

We decided to survey in a kindergarten setting as it is the easiest way to access parents, and to administer the context specific questionnaire. In addition, kindergartens were chosen as a setting, keeping in mind that in the context of a hospital and pharmacy, parents are often under pressure due to medical problems of the child, and do not have enough time to fill-in the questionnaire. 

### 2.2. Data Collection

From March 2016 to October 2016 in Belgrade, Serbia, the survey was conducted in 10 kindergartens in a few municipalities of the city of Belgrade, Serbia. Respondents gave informed consent to participate in the study. All data were collected and analyzed anonymously, in order to keep the privacy of the respondents, as stated in the procedure approved by the Committee for Biomedical Research Faculty of Pharmacy, Belgrade (321/2, 15 March 2016).

Parents had to complete: (a) the previously validated instrument for health literacy (Short Test of Functional Health literacy in adults, S-TOFHLA [21,22,27] (Serbian version), (b) the Pharmacotherapy literacy questionnaire in Serbian (PTHL-SR questionnaire) [24], and (c) a socio-demographic questionnaire which included questions about age, sex, education, employment status, self-estimation of health condition, the number of annual visits to pediatrician, presence of chronic diseases of child, breast feeding information and whether parents were smokers or not. Participants in the survey did not receive any monetary compensation.

The questionnaire was distributed by an interviewer (SU) who was trained to describe necessary information about the survey and research. A printed survey was offered and administered to parents at the scheduled regular parent-teacher meetings in the kindergarten. Parents were allowed unlimited time to complete the PTHL-SR and socio-demographic questionnaire, and 7 min for completion of S-TOFHLA (Serbian version). 

### 2.3. Study Instrument

Health literacy was examined by S-TOFHLA as a continuous variable. It calculated the number of correct answers for each of 36 items in S-TOFHLA, for every participant in survey, in order to understand whether they have inadequate (≤16 correctly answered questions), marginal (17–22) or adequate (≥23) health literacy.

The PTHL-SR questionnaire included 14 questions and was developed and validated in our previous study [24]. Some of the questions included graphic presentations of a measuring device for dosage of liquid pediatric medicines and graphical presentation of packaging for medicines. The graphic images were used as the aim of the study was it be carried out without the active participation of interviewers. Parents were excluded if they had vision problems or reported that they felt sick. Questions in PTHL-SR questionnaire encompassed four domains of pharmacotherapy literacy: knowledge, understanding, numeracy and access to medicine related information.

An overall score in PTHL-SR for each participant was calculated as the percentage of correct answers. In order to examine the influence of socio-demographic characteristics of parents on different levels of pharmacotherapy literacy we divided total scores in the PTHL-SR into three clusters according to terciles. The first cluster were scores with up to 8 (64%) correct answers (low total score in PTHL-SR), the second were scores between 9 and 10 (65–85%) of correct answers (medium score), and the third cluster were score results between 11 and 14 (86–100%) of correct answers (high score). 

The percentage of correct answers for each question was calculated, and the percentage of correct answers within domains of pharmacotherapy literacy. 

### 2.4. Statistical Analysis

Statistical testing of group differences for categorical variables was examined by the chi-squared test of independence. Calculated scores for knowledge, understanding of information, numeracy skills and total PTHL-SR were compared between the groups by Student’s *t*-test for two samples and one way analysis of variance (ANOVA) with post hoc Tuckey-Kremer test. Using binary univariate and multivariate logistic regression analysis we determined the probability of socio-demographic characteristics to predict low PTHL-SR score. All calculations were performed using SPSS, version 22.0 (IBM Corp., Armonk, NY, USA).

## 3. Results

In total 1200 questionnaires were given to parents in kindergartens, and 856 were collected (71.33% response rate). Most of parents said that their reason for non-response was a lack of time. After excluding questionnaires with no or double answers, 813 questionnaires were analyzed.

The present study was context specific, performed outside of medical settings, (i.e., in kindergartens) to minimize pressure related to children’s health conditions. In addition, we have used the S-TOFHLA test, designed to provide quick estimation of health literacy in medical or educational setting (kindergarten).

We found that 99% of parents had adequate functional health literacy assessed by S-TOFHLA (mean score was 33). Mean score of PTHL-SR was 72.83% (standard deviation was 13.37%). Mean PTHL-SR score is the percent of questions answered correctly. Only 25% of parents had less than 90% correct answers in S-TOFHLA, while 75% of parents had less than 85% correct answers in PTHL-SR questionnaire. Our survey showed that 21.5% of parents with adequate health literacy achieved by S-TOFHLA, had the lowest level of pharmacotherapy literacy achieved in PTHL-SR questionnaire. 

All examined parents characteristics are summarized in Table 1. Most of the participants were women (81.30%), who were married (90.2%), between 31 and 40 years old (70.85%), with two children (56.70%). Only 9.8% were single parents (widows, divorced, parents without partners). Every fifth participant estimated their health condition as excellent. Most of respondents (56.6%) had a university degree (at least 16 years of education), 88.1% were employed and were non-smokers (70.2%). In addition to this, our results showed that 80% of parents are reading Patients leaflet instructions before use of medicines. Those who read are mostly non-smokers (71.9%) and parents with two and more children (57%). 

More than 90% of parents recognized the medicine shown in the picture, answered correctly the numerical skills questions and knew to mark an exact dose with a measuring device (97.7%, 94.8%, 96.3%, respectively). In addition, 94.7% of parents would seek information about medicines from a doctor or pharmacist and 94.8% of parents could correctly dose a liquid pediatric medicine using an oral syringe. Conversely, only 38.7% of parents would make a dosage to the child based on their weight and not on their age and only 43.3% were able to interpret the paracetamol dosage chart written on package. On other questions most of the parents answered correctly. Most of responders were able to correctly calculate the dose of oral syrup for a child based on a dosage regimen per kg, knew to correctly interpret the warning statement “Avoid sun while taking this medicine” and knew the appropriate usage of OTC medicine for the children (57.2%, 64% and 76.5%, respectively). 79.3% of parents correctly understood the warning statement about avoiding milk while taking medicine and 84.4% of parents would not give an aspirin to the pre-school children (neither to children up to 16 years). The list of questions and correct answers in PTHL-SR were shown in Table 2.

Parent’s pharmacotherapy literacy expressed as a score per each domain (knowledge, understanding information, numeracy) and total PTHL-SR score was compared according to socio-demographic characteristics. A higher knowledge score was seen in females, married parents, in parents with university degrees and higher education and in non-smokers.

In addition, knowledge scores were different between age groups. The youngest parents had significantly lower knowledge compared to parents in the group of 31–40 years old (*p* = 0.007) and parents aged 41–50 (*p* < 0.001). Married and higher educated responders showed higher scores for understanding of information compared to single responders and responders with middle school degrees. Scores for numeracy skills were higher in responders with University degree, in employed and non-smoking responders. Moreover, ages influenced numeracy skills score. The youngest parents had significantly lower numeracy skills compared to parents in the group of 31–40 years old (*p* = 0.002) and among parents aged 41–50 (*p* = 0.027). Higher performance of the total PTHL-SR score was shown in females, married parents, in parents with University degrees and higher education and in non-smokers. Parents with more than two children had higher total PTHL-SR scores than parents with one child (*p* = 0.016). Youngest responders had lower total PTHL-SR score than responders in the second and third groups (*p* < 0.001 for the both comparisons). Results of parent’s pharmacotherapy literacy according to socio-demographic characteristics are presented in Table 3.

We divided total PTHL-SR score into three clusters (low, medium and high) and examined dependence of different levels of pharmacotherapy literacy on the socio-demographic characteristics of parents (Table 4). The large number of male responders (42.1%) was in the group with low pharmacotherapy level while only 28% females were in the same group (*p* < 0.003). One third of parents (33%) with highest educational grade (faculty and PhD studies) had the highest scores in PTHL-SR, but at the same time only 16% of parents with a lower level of education had the highest scores (*p* < 0.001). Furthermore, 42.5% of single parents (divorced, widows, parents without partners) were in the group with the lowest PTHL-SR scores, while only 29.3% of parents who are married or living in common-law were in the same group (*p* = 0.044). Responders who had excellent self-estimation of health status and non-smokers were in the majority in the high total score group, and in the minority in low total score group (*p* = 0.048 and *p* = 0.001, respectively). Dependance of total PTHL-SR score on socio-demographic characteristics is shown in Table 4.

Only 17.8% of males compared to 82.2% of females would ask a doctor or pharmacist for information about which medicines to give to a child for pain relief and fever. Other parents would search for information from different sources (e.g., neighbors, internet, newspapers). This finding suggests that broad access to information via INTERNET or newspapers are common among parents with lower health literacy, especially males. 

Parents characteristics associated with lower PTHL-SR results are presented in Table 5. Men had almost 2 times higher probability for low PTHL-SR scores then women (OR 1.871; *p* < 0.001). Older parents had lower probability for low scores−parents older than 30 years had less than half probability for low scores (OR 0.500 for parents from 30 to 40 years and OR 0.460 for parents from 41 to 50 years) compared to parents younger than 30 years. Furthermore, the number of children in a family was associated with low PTHL-SR score; if number of children was higher probability for low total PTHL score was lower. Single parents had almost two times higher probability of low scores than married ones (OR 1.781, *p* < 0.016). Higher education and excellent self-estimation of health are less likely to achieve low total scores in PTHL-SR (OR 0.450 and OR 0.533, respectively). 

Furthermore, we determined independent predictors of low PTHL-SR scores after inclusion of significant socio-demographic characteristics in the logistic model. Significant independent predictors of the low PTHL-SR score were male gender, high education and number of children. Male gender was associated with higher probability [OR—1.79, 95% CI (1.239–2.588), *p* = 0.002] for low total score. High education [OR—0.471, 95% CI (0.349–0.636), *p* < 0.001] and higher number of children were associated with lower probability for low total score [OR for parents with two children −0.701, 95% CI (0.504–0.975), *p* = 0.035 and OR for parents with more than two children was 0.591 95% CI (0.356–0.981), *p* = 0.042]. 

## 4. Discussion

This is the first specific study that examines pharmacotherapy literacy of parents related to use of medicines for pre-school children in Serbia. We found that many parents of pre-school children in Serbia do not accurately understand basic information about medicines for their children, regardless whether the information is written or spoken.

Our results show that more than half of parents (56.7%) could not properly understand dosage regimen of OTC medicines written on the package and in Patient Information leaflets, especially warning statements (for example, about avoiding sun and milk during medical therapy). Previous study among pharmacy visitors in Australia [5], found that auxiliary label statements were misinterpreted among a majority of responders. Findings from the study by Emmerton et al. [11] suggested that half of parents could not accurately determine weight-based doses. In our study, almost two/third of parents would make dosage based on the age and not on the weight of the child, and every 4th parent did not know appropriate use of OTC medicine for children. 

This indicates that information about medicines should be written in a simpler way [28] or that the majority of parents need more clarification from a pharmacist or pediatrician. Writing of information about medicines use in a simpler way and ease of communication between parents and healthcare professionals are the key measures that should be done in order to facilitate the use of medicines by parents in cases of acute illness or preventive care. These measures could also include visual aids for those with communication barriers [29], and the necessity to check if parents understood information of medicines [19], by determining if parents can explain information with their own words.

In our study, higher scores of PTHL-SR closely correlated with higher educational level, female sex, married parents, parents with more children, parents who estimated their health condition as excellent, non-smokers and those who visit pediatricians less times per year. 

This study showed that parents with higher education level (university degree or higher with more than 16 years of education) had significantly better overall score in PTHL-SR as well as in knowledge, understanding and numeracy. The results are in line with previous studies on health literacy [13,15,16,17,18,20].

A previous study on health literacy in Serbia [22] highlighted that there is no significant difference in functional health literacy level between males and females. Our results highlight gender differences in pharmacotherapy literacy in the tested population. While 42% of men had the lowest PTHL-SR score, this was true for only 28% of women. Moreover, women had better scores in all defined domains: knowledge, understanding and numeracy in comparison to men (*p* < 0.001). This might be because women as mothers are predominantly taking care of ill children and because our survey responders were mostly women.

In general, older parents (41–50 years old) had the highest mean score in knowledge about pediatric medicines (4.04 ± 0.92, *p* < 0.001). This can be explained by the empirical knowledge about medicines use by older parents. 

Our findings on parents with more than two children having better scores for knowledge and total PTHL-SR score, may also be related to previous experience and empirical knowledge gained during care and medication of children. This finding implies that healthcare professionals, especially pharmacists, could actively assist in helping younger parents who have one child, firstly in clarifying given information by repeating instructions. Parents have to be encouraged to become more educated, either by self-education through reliable sources of information or through educational campaigns organized by healthcare professionals. Interestingly, our study also showed that single parents are more likely to have lower pharmacotherapy literacy, especially in knowledge and understanding of medicines related information. This could be explained by the lack of mutual interaction when people can influence each other’s behavior and could learn from each other.

Our study results confirmed findings from previous studies of health literacy [22] that people who estimated their health condition as excellent had an adequate functional health literacy, as well as the highest (26.3% of parents) overall scores measured by PTHL-SR questionnaire. 

Furthermore, parents who smoke showed decreases overall scores in PTHL-SR together with limited knowledge and numerical skills. This correlates with a previous study conducted by Radic et al. in Serbia [30], where smoking was significantly frequent among less educated parents, both mothers and fathers.

Results of the PTHL-SR illustrated some more important facts. Every fourth parent in Serbia has difficulty in understanding common information written on the package or in Patient Information leaflets, especially warning statements (for example, about avoiding sun and milk during medical therapy). About 20% of parents do not read patient leaflet instructions, but rather rely on advice from a pharmacist or pediatrician.

Data presented showed that adequate health literacy results, as assessed by S-TOFHLA, are not an accurate indicator of parental pharmacotherapy literacy, especially in parents with a higher level of education. 

This is primarily because the PTHL-SR tests understanding, knowledge, access to medicines related information and numeracy, while the S-TOFHLA test examine only reading comprehension and understanding of medical terms. PTHL-SR seems to be a more accurate measure of pharmacotherapy literacy among parents of pre-school children.

The results from our study emphasize the need for evaluation of medication labelling in order of improving instructions about pediatric medicines use, especially for parents with limited health and pharmacotherapy literacy. It is also a trigger to improve communication of health care professionals, particularly pharmacists, with aim to reduce errors in medication of pediatric patients made by parents. 

### Limitations

Certain limitations of the present study should be taken into consideration when interpreting results. As this is a cross-sectional study, we didn’t correlate the PTHL-SR with clinical outcomes. However, the study shows that parents of pre-school children in Serbia have problems in appropriate dosing and understanding information about use of pediatric medicines.

The study does not investigate causes of low parental pharmacotherapy literacy, just correlation of low PTHL-SR results with socio-demographic characteristics of parents. Moreover, the study is done in an urban area, among parents with higher education degree. Future studies should consider parents outside urban area and further improvement of the PTHL-SR reliability and feasibility.

## 5. Conclusions

These results provide evidence that limitations in understanding common information about use of medicines are widespread among parents of pre-school children and encourage efforts for further investigation. The PTHL-SR questionnaire may be a useful tool for identification of parents who need more instructions and assistance from healthcare providers, above all in providing better written or spoken information at community pharmacy settings.

## Figures and Tables

**Table 1:** Socio-demographic characteristics of the parents in the survey.

Socio-Demographic Characteristics		No.	%
Sex	Male	152	18.7
	Female	661	81.3
Age (years)	18–29	59	7.26
	30–40	576	70.8
	41–50	161	19.8
	51–60	17	2.09
Number of children	One child	245	30.1
	Two children	461	56.7
	Three and more children	107	13.1
Marital state	Single parent ^a^	80	9.8
	Married/Common-law	733	90.2
Education	University degree and higher ^b^	460	56.6
	Middle school or less ^c^	353	43.3
Employment	Employed	716	88.1
	Unemployed	97	11.9
Self-estimation of health status	Average and Bad	144	17.7
	Good	502	61.7
	Excellent	167	20.5
Chronic diseases	No	710	87.3
	Yes	103	12.7
Smoker	No	570	70.2
	Yes	243	29.8
Breast feeding of a first child	No	85	10.5
	Yes	728	89.5
Annual visits to pediatrician	1–2 times a year	270	33.2
	3–4 times	263	32.3
	5–6 times a year and more	280	34.5

In Table 1 are presented absolute and relative frequences. ^a^ Single parents (widows, divorced, living with a child alone). ^b^ University degee and higher (at least 16 years of education). ^c^ Middle school or less (8–12 years of education).

**Table 2:** List of questions and percent of correct answers in PTHL-SR questionnaire.

No	Question	Domain	% Correct
1	What is this medicine (ibuprofen) used for?	Knowledge	76.5
2	What does this medicine contain? (picture of paracetamol syrup)	Knowledge	97.7
3	Would you give an aspirin to a child of 6 years if it has a fever?	Knowledge	84.4
4	Your child has otitis and pain. How do you calculate the dose for a child/Where do you find information how much medicine for pain relief to give (per kg or per age)?	Knowledge	38.7
5	What is the highest temperature limit after you give antipyretic to a child?	Knowledge	89.3
6	Pharmacist told you to avoid milk and milk products while taking medicine. What does it mean to you?	Understanding	79.3
7	Avoid sun while taking medicine. What does it mean to you?	Understanding	64.0
8	Keep under 25 °C. After reconstitution, keep refrigerated up to 14 days. How will you store this medicine after reconstitution?	Understanding	83.4
9	You have to give medicine to a child 2 times a day. If your package has 10 items, how many medicines you will have after 3 days?	Numeracy	94.8
10	To mark the dosage for a child of 13 kg on measuring spoon.	Numeracy	96.3
11	To answer how much medicine is inside the oral syringe.	Numeracy	92.6
12	To calculate a dose of oral syrup for child based on dosage regimen per kg.	Numeracy	57.2
13	To interpret paracetamol dosage chart written on package, per weight.	Numeracy	43.3
14	Where did you get an information how much antipyretic to give to your child?	Access	94.7

**Table 3:** Parent’s pharmacotherapy literacy according to socio-demographic characteristics.

Parents Characteristics	Knowledge X ± SD (Max Score: 5)	*p* Value	Understanding X ± SD (Max Score: 3)	*p* Value	Numeracy X ± SD (Max Score: 5)	*p* Value	Total Score X ± SD (Max Score: 14)	*p* Value
Sex								
Male	3.64 ± 1.02	<0.001	2.41 ± 0.89	0.243	3.81 ± 0.98	0.693	9.88 ± 2.06	0.019
Female	3.91 ± 0.88		2.50 ± 0.85		3.85 ± 0.91		10.27 ± 1.82	
Age (years)								
18–29	3.46 ± 1.21	<0.001	2.25 ± 0.97	0.105	3.44 ± 1.04	0.004	9.15 ± 2.43	<0.001
30–40	3.86 ± 0.87 *		2.53 ± 0.85		3.89 ± 0.89 *		10.28 ± 1.78 *	
41–50	4.04 ± 0.92 *		2.44 ± 0.86		3.83 ± 0.98 *		10.32 ± 1.86 *	
51–60	3.76 ± 0.83		2.41 ± 0.79		3.71 ± 0.98		9.88 ± 1.87	
Number of children								
One child	3.75 ± 0.98	0.050	2.41 ± 0.93	0.118	3.78 ± 0.94	0.057	9.94 ± 2.01	0.014
Two children	3.92 ± 0.89		2.50 ± 0.84		3.83 ± 0.92		10.25 ± 1.79	
Three children and more	3.89 ± 0.88		2.61 ± 0.81		4.04 ± 0.90		10.54 ± 1.83 *	
Marital status								
Single parent	3.61 ± 1.23	0.009	2.23 ± 0.99	0.006	3.86 ± 0.91	0.067	9.51 ± 2.50	<0.001
Married	3.89 ± 0.87		2.51 ± 0.85		3.66 ± 1.04		10.27 ± 1.78	
Education								
University degree and higher ^a^	3.98 ± 0.82	<0.001	2.60 ± 0.83	<0.001	3.99 ± 0.86	<0.001	10.58 ± 1.67	<0.001
Middle school and less education ^b^	3.71 ± 1.00		2.34 ± 0.89		3.64 ± 0.97		9.69 ± 1.99	
Employment								
Employed	4.01 ± 0.94	0.098	2.50 ± 0.85	0.122	3.87 ± 0.89	0.021^*^	10.22 ± 1.79	0.296
Unemployed	3.84 ± 0.91		2.36 ± 0.96		3.64 ± 1.14		10.01 ± 2.37	
Self-estimation of health status								
Average and bad	3.85 ± 1.00	0.557	2.43 ± 0.88	0.299	3.71 ± 1.06	0.080	10.00 ± 2.26	0.079
Good	3.85 ± 0.91		2.47 ± 0.89		3.84 ± 0.91		10.17 ± 1.84	
Excellent	3.93 ± 0.84		2.57 ± 0.76		3.95 ± 0.85		10.46 ± 1.60	
Chronic diseases								
No	3.84 ± 0.92	0.089	2.48 ± 0.88	0.652	3.84 ± 0.94	0.553	11.94 ± 1.97	0.101
Yes	4.01 ± 0.91		2.52 ± 0.77		3.89 ± 0.84		12.27 ± 1.71	
Smoking								
No	3.91 ± 0.89	0.023	2.50 ± 0.85	0.573	3.91 ± 0.88	<0.001	10.33 ± 1.75	0.002^*^
Yes	3.75 ± 0.96		2.46 ± 0.91		3.66 ± 1.01		9.88 ± 2.10	
Breast feeding of a first child								
Yes	3.88 ± 0.90	0.845	2.49 ± 0.84	0.945	3.90 ± 0.94	0.129	10.27 ± 1.88	0.520
No	3.82 ± 0.93		2.46 ± 0.96		3.88 ± 0.84		10.16 ± 1.98	
Annual visits to pediatrician								
1–2 times	3.90 ± 0.88	0.447	2.53 ± 0.90	0.770	3.88 ± 0.92	0.579	10.31 ± 1.83	0.352
3–4 times	3.89 ± 0.92		2.48 ± 0.87		3.86 ± 0.93		10.23 ± 1.92	
5–6 times and more	3.83 ± 0.94		2.45 ± 0.83		3.79 ± 0.93		10.07 ± 1.86	

Groups were compared by Student *t* test for two samples or one-way ANOVA test. * Significant difference between groups (*p* ≤ 0.05), determined by Post Hoc Tukey-Kramer test. ^a^ University degree and higher (at least 16 years of education). ^b^ Middle school or less (8–12 years of education).

**Table 4:** Dependance of total PTHL-SR score from socio-demographic characteristics.

Parents Characteristics	Total Score			*p* Value
	Low	Medium	High	
Sex				
Male	42.1%	36.2%	21.7%	0.003
Female	28.0%	45.5%	26.5%	
Age (years)				
18–29	45.8%	42.4%	11.9%	0.07
30–40	29.7%	43.4%	26.9%	
41–50	28.0%	44.7%	27.3%	
51–60	35.3%	52.9%	11.8%	
Number of children				
One child	37.1%	40.0%	22.9%	0.082
Two children	28.9%	44.7%	26.5%	
Three children and more	23.4%	48.6%	28.0%	
Marital status				
Single parent	42.5%	33.8%	23.8%	0.044
Married	29.3%	44.9%	25.8%	
Education				
University degree and higher ^a^	23.3%	43.7%	33.0%	<0.001
Middle school and less (8–12 years of education) ^b^	40.2%	43.9%	15.9%	
Employment				
Employed	30.9%	43.6%	25.6%	0.916
Unemployed	28.9%	45.4%	25.8%	
Self-estimation of health status				
Average and bad	34.0%	43.1%	22.9%	0.048
Good	32.7%	41.2%	26.1%	
Excellent	21.6%	52.1%	26.3%	
Chronic diseases				
No	31.3%	43.2%	25.5%	0.562
Yes	26.2%	47.6%	26.2%	
Smoker				
No	30.9%	43.5%	25.6%	0.001
Yes	35.5%	42.1%	22.3%	
Breast feeding of a first child				
Yes	30.8%	42.6%	26.8%	0.912
No	28.2%	45.9%	25.9%	
Annual visits to pediatrician				
1–2 times	32.2%	38.5%	29.3%	0.266
3–4 times	27.4%	47.1%	25.5%	
5–6 times and more	32.1%	45.7%	22.2%	

^a^ University degree and higher (at least 16 years of education). ^b^ Middle school or less (8–12 years of education).

**Table 5:** Parent’s characteristics associated with lower PTHL-SR results.

Characteristic	OR	95% CI	*p* Value
Sex			
Male sex	1.871	1.300–2.693	<0.001
Age (years)			
30–40	0.500	0.291–0.861	0.012
41–50	0.460	0.248–0.852	0.014
Number of children			
2 children	0.686	0.494–0.953	0.025
3 children	0.516	0.308–0.866	0.012
Marital status			
Single	1.781	1.112–2.852	0.016
Married (Common-law)			
Education			
Higher education	0.450	0.333–0.610	<0.001
Self-estimation of health status			
Excellent	0.533	0.322–0.883	0.015
